# Revisiting Pulmonary Sclerosing Pneumocytoma

**DOI:** 10.3390/clinpract14040116

**Published:** 2024-07-22

**Authors:** Claudia Manini, Simone Vezzini, Antonella Conte, Giuseppe Sciacca, Alessandro Infantino, Poliana Santos-Pereira, José I. López

**Affiliations:** 1 Department of Pathology, San Giovanni Bosco Hospital, ASL Città di Torino, 10154 Turin, Italy; claudia.manini@aslcittaditorino.it (C.M.); poliana.santospereira@aslcittaditorino.it (P.S.-P.); 2 Department of Sciences of Public Health and Pediatrics, University of Turin, 10124 Turin, Italy; 3 Pathology Unit, Department of Medical Sciences, University of Turin, 10126 Turin, Italy; simone.vezzini@unito.it (S.V.); antonella.conte@unito.it (A.C.); giuseppe.sciacca@unito.it (G.S.); 4 Faculty of Medicine and Dentistry, University of Rome “La Sapienza”, 00185 Rome, Italy; infantino.2093289@studenti.uniroma1.it; 5 Biobizkaia Health Research Institute, 48903 Barakaldo, Spain

**Keywords:** pulmonary sclerosing pneumocytoma, pulmonary neuroendocrine hyperplasia, well-differentiated neuroendocrine tumor, lung adenocarcinoma, differential diagnosis, immunohistochemistry

## Abstract

Pulmonary sclerosing pneumocytoma (PSP) is a quite rare tumor outside Eastern countries. This rarity, together with a wide histological appearance, makes its correct identification a diagnostic challenge for pathologists under the microscope. Historically, PSP was considered a vascular-derived neoplasm (sclerosing hemangioma), but its immunohistochemical profile clearly supports its epithelial origin. No specific molecular fingerprint has been detected so far. This short narrative revisits the clinical, histological, immunohistochemical, and molecular aspects of this tumor, paying special attention to some controversial points still not well clarified, i.e., clinical aggressiveness and metastatic spread, multifocality, the supposed development of sarcomatoid change in a subset of cases, and tumor associations with lung adenocarcinoma and/or well-differentiated neuroendocrine hyperplasia/tumors. The specific diagnostic difficulties on fine-needle aspiration cytology/biopsy and perioperative frozen sections are also highlighted. Finally, a teaching case of tumor concurrence of lung adenocarcinoma, neuroendocrine lesions, and PSP, paradigmatic of tumor association in this context, is also presented.

## 1. Introduction

Pulmonary sclerosing pneumocytoma (PSP), formerly sclerosing hemangioma [1], is a rare pulmonary tumor with a varied histological morphology [2,3,4,5,6], which makes its correct diagnosis a challenging task in some practical situations [7,8]. This tumor usually presents as a solitary nodule sometimes mimicking pulmonary carcinoma [9]; however, multiple PSPs have also been reported [10,11,12,13]. Although traditionally considered a benign tumor, some recent series have detected malignant features at least in a subset of cases [14], including sarcomatoid dedifferentiation [6], local recurrence [15,16], and metastatic seed [4,5,17]. Interestingly, a relevant number of these tumors are coincidental with pulmonary neuroendocrine-related tumors [10,12,18,19,20,21,22,23], although the probable pathogenic links connecting these two conditions still remain under discussion. Recent studies have analyzed the genomic and transcriptomic profile of PSP [11,24,25], trying to shed some light on the tumor etiopathogenesis.

This review revisits the clinical presentation and the varied histological spectrum of this entity, calls for the attention of pathologists in considering this diagnosis in small core biopsies or in intraoperative frozen sections, and focuses on several unanswered questions, for example, stromal overgrowth and possible sarcomatoid dedifferentiation, tumor multifocality/bilaterality, tumor aggressiveness and potential malignancy, and association with other lung tumors. As an example, a typical case of PSP associated with a well-differentiated neuroendocrine tumor and lung adenocarcinoma is added at the end to illustrate these pathological features and clinical associations.

## 2. Material and Methods

The authors have scrutinized the PubMed database, considering the following keywords: sclerosing hemangioma, sclerosing pneumocytoma, and pulmonary pseudotumor. Since this entity is quite rare in Western countries, the goal of this review is to make PSP familiar to clinicians, radiologists, and pathologists in a single narrative. For such a purpose, published articles in English in the last five years focusing on clinical-radiological data, histopathological findings, and tumor associations have been included in the list of references, as well as classic descriptions with historical significance.

## 3. Clinical Features

PSP affects most frequently women in Eastern countries. Series from China [3,4,5,18,26,27], Taiwan [8], Japan [15], Korea [28], and the USA [2] confirm female predominance, the wide age range of patients, no clear relationship with smoking, more frequent involvement of lower lobes, and a predominant clinical presentation as a solitary nodule. Cases with multiple nodules, however, even bilateral, have also been reported [10,11,12,13]. Most of the reported cases show a benign clinical course, but aggressive examples, including cases with distant metastases, also occur [4,5,6,14,15,16,17]. Wang et al. [5] have detected significant differences in gender, age, and tumor size in PSP with and without metastases. Location within the lung seems to matter. For example, Zhang et al. [27] have detected significant differences in multiphase CT scans between central- vs. peripheral-located tumors within the lung in 33 patients (all female), serving as helpful tools in distinguishing the nature of borderline tumors.

The most important clinical and radiological differential diagnosis of PSP is lung cancer. In fact, PSP may mimic cancer both clinically and radiologically; indeed, it can be associated with it, either cancer and PSP taking part of the same nodule [29] or as separated nodules [20,30,31]. A recent case from our own experience in which PSP, lung adenocarcinoma, and well-differentiated neuroendocrine tumors meet illustrates this frequent practical clinical situation (see later).

Considering that lung cancer or solitary lung metastasis is the first diagnostic option, most PSPs are diagnosed in different types of lung resections depending on its size, location, and clinical context.

## 4. Histopathological Diversity

Early microscopic descriptions considered PSP an endothelial-derived benign neoplasm [1]. For years, the true nature of the cell of origin of this tumor remained under discussion. Aside from endothelial derivation [32], mesothelial [33], mesenchymal [34], neuroendocrine [35], and even epithelial [36] origins were also considered. Despite such varied proposed origins, the name pulmonary sclerosing hemangioma gained momentum at that time and still appears in many relatively recent reports of this tumor [23]. The epithelial nature of PSP was definitely proved when TTF-1 immunoreactivity was detected in both surface and round cells in the largest series published so far [2].

On light microscopy, PSP displays a somewhat confusing morphology, with different cells and architectural patterns taking active part in the tumor (Table 1). Interestingly, Nagata et al. [36] quite correctly pointed out already in 1987 that PSP was an epithelial tumor composed of two cell types. The tumor is in fact composed of two cell types (cuboidal surface cells and stromal round/polygonal cells) and may present different growth patterns, i.e., solid, sclerotic, papillary, and hemorrhagic [2,4]. Associations of different growth patterns within the same tumor are frequently seen. So, some cases present a solid pattern of growth with scattered sclerotic foci. Other cases show a predominant papillary pattern with uniform cuboidal cells, often with multinucleation, intranuclear inclusions, and vacuolated cytoplasm. Still some others display prominent sclerosis, with dense hyaline collagen bands around hemorrhagic areas. Finally, some PSPs present predominantly hemorrhagic patterns showing abundant hemosiderin pigment and foamy histiocytes within the tumor. True atypia is rare or absent. A recent series of 68 cases shows that most cases (85.4%) included two or more of these histological patterns within the tumor [5]. This broad spectrum of possible morphologies is a reason for many diagnostic misinterpretations, especially in core biopsy specimens, in cytologic material obtained by endobronchial ultrasound-guided transbronchial needle aspiration, and in frozen sections. Such challenging diagnostic situations are considered later in a specific section.

## 5. Immunohistochemical and Molecular Findings

Based on its consistent TTF-1 positivity (Table 1), Devouassoux-Shisheboran et al. [2] already in 2000 suggested that PSP was a neoplasm most probably derived from the primitive respiratory epithelium and not of an endothelial origin as previously believed. The immunohistochemical expression is coincident with that of type 2 pneumocytes. Aside from TTF-1 positivity, the epithelial (surface) cells show epithelial membrane antigen (EMA), napsin A, and cytokeratin 7 (CK7) positivity, while the stromal component is negative with napsin A. The different immunohistochemical profile of surface and stromal cells have led researchers to consider that stromal cells represent the true neoplastic component while the surface cells are reactive type 2 pneumocytes [37].

Although a minority of PSPs may show positive immunostaining with BRAF V600E, none of them present the mutation of this gene by PCR analysis [5]. A recent next-generation sequencing study has revealed AKT1 internal tandem duplications, point mutations, and short indels in practically all tested cases [24,25], but the clinical and prognostic significance of these findings remains unknown. PI3K/AKT/mTOR pathway activation has also been detected [11,24,25], as well as SOX9 upregulation [25], in a significant number of PSPs. *AKT1* gene mutations have been detected in up to 78% of the analyzed cases, as reported by Boland et al. in a series of 10 cases [24]. In addition, Fan et al. [11] have detected somatic mutations in 15 genes (*MEGF6*, *DNAH5*, *AKT1*, *GPRIN2*, *PIK3AP1*, *FBXO40*, *HERC1*, *VPS16*, *MORN1*, *ZNF474*, *CTNNB1*, *ZNF251*, *TSC1*, *ATM*, *KDR*).

## 6. Diagnostic Walks on the Tight Rope

Radiologically guided core biopsies are common procedures in daily hospital practice. CT scan-guided and fine-needle aspiration biopsy offer high accuracy in approaching pulmonary lesions, with high diagnostic performance and low rates of secondary effects, even in small nodules [38,39,40]. Their usefulness lies in the rapid confirmation of cancer diagnosis, primary or metastatic, offering tissue material for molecular analysis. However, there is always a potential problem of tumor representativeness, a fact of paramount importance in some instances of high intratumor heterogeneity. Aside from detecting the different subtypes of lung cancer, the differential diagnosis of CT scan core biopsies also includes pulmonary pseudotumors, infections, and benign tumors, for example, PSP.

PSP is a very infrequent tumor outside Eastern countries and shows different architectural patterns with two cell types participating in it. Under these conditions, its diagnosis may be really difficult especially if the obtained material is scarce or the tumor is not optimally sampled. Several case reports of PSPs diagnosed using this tool appear in the literature [41,42,43,44]. Some of these authors report diagnostic difficulties using core biopsy specimens. For example, Morikawa et al. [41] describe a case of PSP presenting with multiple nodules in which the diagnosis of lung adenocarcinoma was considered based on its papillary architecture.

Fine-needle aspiration cytology (FNAC) has been occasionally used as the initial step to diagnose PSP [45,46,47,48,49]. Most authors confirm that the cytological diagnosis of this tumor is difficult [46,47,48] and some of them advise to not rest such diagnosis only on cytological data. Saha et al. [45] report a case studied on material obtained by CT-guided FNAC where PSP cells were interpreted as well-differentiated adenocarcinoma of the lung. Based on this preliminary report, the nodule was surgically removed and the correct diagnosis was performed on histology. Hissong et al. [48] and Kosmas et al. [49] also include carcinoid tumors in the cytological differential diagnosis of PSP.

The diagnostic challenge of PSP in intraoperative frozen section material has been recently reviewed in two large series from China [7] and Taiwan [8]. Shang et al. [7] describe a significant difference in the percentage of intraoperative misdiagnosis depending on the PSP size (≤1 cm vs. >1 cm), with up to 11% of misdiagnoses in small (≤1 cm) cases. Interestingly, the presence of solid and papillary growth patterns were the main factors of diagnostic error. On the other hand, Yang et al. [8] report a diagnostic accuracy of only 44% of their 59 intraoperatively analyzed cases. The main pitfalls were provoked by solid architecture, hypercellularity, glandular spaces, sclerosis, atypia, and coagulative necrosis. Cases presenting only with a single histological pattern and/or cytological atypia are particularly problematic to be correctly identified [50].

## 7. Prognostic Features of Concern

The intrinsic nature of PSP and its clinical implications shows several intriguing grey zones. For example, the correlation between stromal overgrowth and/or sarcomatoid-appearing areas, the expected malignant (?) behavior of such a change, and the clinical impact and biological significance of multifocality and metastatic seed still remain under discussion.

Some PSPs show areas composed of spindle-shaped cells with a variable degree of atypia, raising the possibility of malignant transformation. For example, Teng et al. [51] have reported the malignant transformation of both surface and stromal cells in a PSP with multiple lymph node metastases. Zhang et al. [14] report a PSP measuring 8.9 cm in diameter containing fibrosclerotic areas and hypothesize that the development of tumor metastases might be associated with stromal overgrowth. Actually, a retrospective study has shown that up to 20% of PSPs with dense stromal overgrowth presented lymph node metastases [4]. More recently, Liang et al. [6] have described a case with overt undifferentiated sarcomatoid features.

These single cases support the idea that a subset of PSPs develop an aggressive potential linked to stromal malignant transformation. Wang et al. [52] have found *AKT1* E17K somatic mutation and *TP53* C176Y germline mutation in a PSP with aggressive behavior. Clinical aggressiveness includes not only lymph node [4,5], bone [17], or liver [51] metastases, but also pleural invasion [22,53]. Despite tumor dissemination, deaths directly caused by this tumor are extremely rare. In fact, only a single case of fatal onset has been reported in the literature so far [54].

Multifocality has been occasionally reported in PSPs [5,10,11,12,13,41,54], and this clinical presentation raises some concern about its malignant potential and the exact etiopathogenesis of this tumor presentation. However, as previously mentioned, fatal PSPs have been very rarely reported [54].

## 8. Tumor Associations

The concurrent association of PSP with lung carcinoid tumors and other neuroendocrine lesions has been repeatedly reported in the literature [10,12,18,19,20,21,22,23,36]. Several of these cases include multifocal PSP presentation, and at least one case shows these two conditions taking place in a single (mixed) collision tumor [23]. On the other hand, cases associated with lung cancer [20,29,32], and bronchial adenoma [21] have been reported as well. To date, only Cho et al. [20] have reported a triple tumor association including PSP, carcinoid tumor, and lung adenocarcinoma.

The abundance of PSP cases coincidentally with neuroendocrine lesions (neuroendocrine hyperplasia and/or carcinoid tumors) is an interesting and not well-answered question that suggests a more than casual association. Wang et al. [10] arrived at this conclusion in their report of a patient with multiple PSPs, carcinoid tumors, and extensive neuroendocrine proliferation. Interestingly, multiple PSPs and multifocal pulmonary neuroendocrine hyperplasia are more frequently observed in women [26,55].

Pulmonary neuroendocrine cells participate in the regulation of blood flow and contribute to the modulation of local immune responses among other important functions. However, the possible nexus between both lesions remains unknown. Some experimental studies [35] have highlighted the important effect of neuroendocrine cells in Clara cells and pneumocyte regeneration after lung injury caused by drugs and viral infections, for example. PSP is thought to have originated from type 2 alveolar cells [4]; however, it is largely unknown if this physiological interplay between neuroendocrine cells and type 2 pneumocytes may promote the development of PSP and neuroendocrine-derived hyperplasia/tumors.

## 9. A Clinical Paradigmatic Example

**Figure 1:**
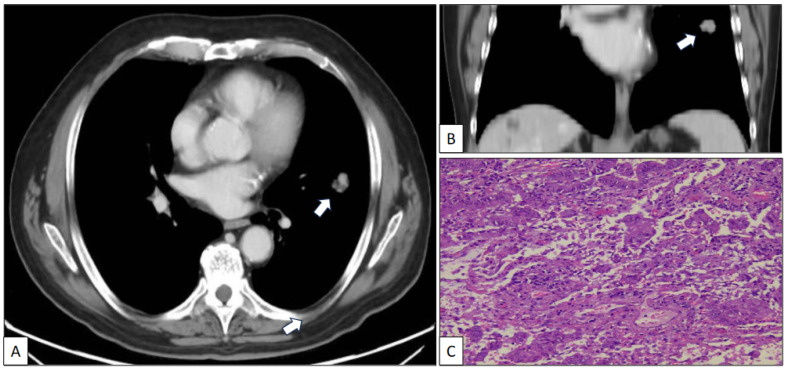
Axial (**A**) and coronal (**B**) CT scan images of a nodule (arrow) in the left upper lobe corresponding to a histologically confirmed pulmonary adenocarcinoma (**C**) (hematoxylin-eosin, original magnification, ×100).

**Figure 2:**
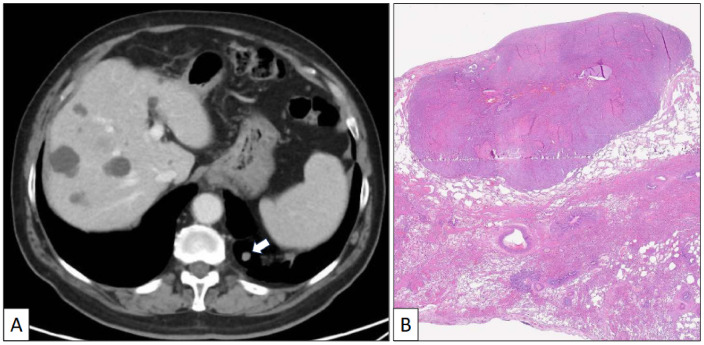
(**A**) Axial CT scan image of a left lower nodule close to the costophrenic angle. (**B**) Histological panoramic view of a well-delimited solid tumor growing beneath the pleural surface (hematoxylin-eosin, original magnification ×1.5).

A 73-year-old man with a previous history of severe tobacco smoking (cessation in 1990) and occupational exposure to toxics (automotive tire manufacturing company) underwent an X-ray and CT scan due to persistent cough and fever. The radiological study revealed a 23 mm in diameter irregular nodule located in the left upper lobe (Figure 1A,B). Additionally, a 10 mm in diameter regular nodule with ventilatory impairment was detected in the left lower lobe close to the costophrenic angle (Figure 2). A fine-needle aspiration (FNA) biopsy of the upper lobe nodule yielded a diagnosis of pulmonary adenocarcinoma. A subsequent PET scan indicated a maximum standardized uptake value (SUV max) of 2.59 in the upper lobe lesion and a faint basal uptake value in the lower lobe lesion. The multidisciplinary consensus advised left upper lobectomy plus atypical lung resection in the left lower lobe. The patient is well and free of disease 1 year after the initial diagnosis.

The left upper lobectomy showed an adenocarcinoma with standard histological features (Figure 1C).

**Figure 3:**
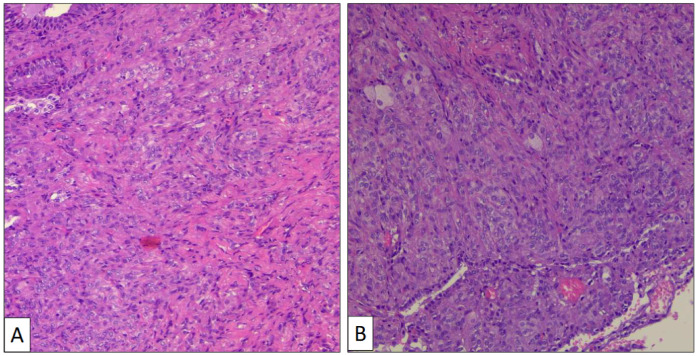
Histological patterns of sclerosing pneumocytoma, including sclerotic (**A**) and solid (**B**) (hematoxylin-eosin, original magnification ×100).

**Figure 4:**
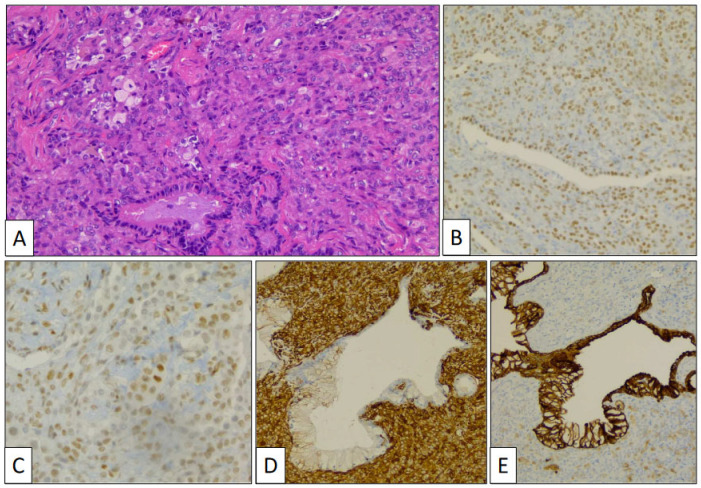
Histological detail of sclerosing pneumocytoma showing stromal and surface cells, and foamy macrophages (**A**) (hematoxylin-eosin, original magnification, ×240). Immunohistochemical study showing positivity with TTF-1 (**B**), FLI-1 (**C**), vimentin (**D**), and AE1/AE3 cytokeratin (**E**).

The atypical left lower lung resection showed a tumor with well-defined limits growing into the lung parenchyma close to the pleura (Figure 2B). The tumor displayed a predominantly dense proliferation of cells with sclerotic (Figure 3A), solid (Figure 3B), and hemorrhagic (Figure 3C) areas. At the tumor periphery, no infiltrative borders were seen and necrosis was not detected. A closer view presented a biphasic cellular pattern with stromal-appearing cells together with surface cells (Figure 4A). No atypia or mitosis was seen. Foamy macrophages were occasionally detected.

Stromal and surface cells were positive with TTF-1 (Figure 4B) and FLI-1 (Figure 4C) markers. AE1/AE3 cytokeratin (Figure 4D) and vimentin (Figure 4E) markers were positive, respectively, in surface and stromal cells. The histological and immunohistochemical findings were conclusive of PSP.

**Figure 5:**
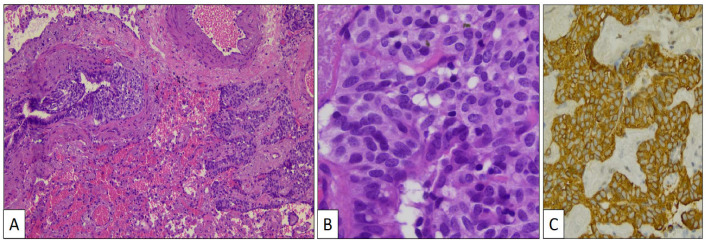
Low-power view of the neuroendocrine proliferation beneath the bronchial epithelium and the infiltrative. component close to pre-existing blood vessels (**A**) (hematoxylin-eosin, original magnification, ×40). High-power view of tumor cells showing neuroendocrine features (**B**) (hematoxylin-eosin, original magnification, ×400) and intense chromogranin immunostaining (**C**).

Aside from the adenocarcinoma in the left upper lobe and the PSP in the left lower lobe, a third lesion was histologically discovered. Small budding nests of monotonous cells growing beneath the normal bronchial epithelium close to cords of similar cells infiltrating the lung perivascular parenchyma (Figure 5A) were specifically detected in the vicinity of the PSP. Cells in both growing patterns showed eosinophilic and finely granular cytoplasm and oval nuclei with salt-and-pepper chromatin characteristic of neuroendocrine differentiation (Figure 5B). This cellular component presented intense chromogranin (Figure 5C) and synaptophysin immunostaining.

## 10. Concluding Remarks

PSP is a tumor, mostly benign in nature, quite rare outside Eastern countries with a proven pulmonary epithelial origin. Although cases with metastases, multifocality, and sarcomatoid transformation do appear, death caused by this disease has been documented in the literature only once. PSP and neuroendocrine-derived lesions, including both hyperplasia and tumors, frequently coexist in the same patients. Associations of PSP with lung carcinoma have been also reported. A spectrum of architectural patterns (solid, sclerotic, papillary, and hemorrhagic), two cell types (surface and stromal), a specific immunohistochemical pattern, and some typical molecular alterations conform the profile of this neoplasm. Due to its varied morphology and its relative rarity, PSP must be always considered in the differential diagnosis of pulmonary nodules, particularly avoiding a misdiagnosis of lung adenocarcinoma. Its diagnosis in small biopsies or frozen sections remains hazardous and a challenge for pathologists, as reported in several recent reports.

## Figures and Tables

**Table 1:** Histologic and immunohistochemical findings in Pulmonary Sclerosing Pneumocytoma.

Architecture	Solid Sclerotic - Dense hyaline bands Papillary Hemorrhagic - Hemosiderin plugs - Foamy histiocytes Mixed (the most frequent, combining two or more of the above)
Cell types	Stromal cells (round/polygonal) Cuboidal cells (surface) - Multinucleation - Intranuclear inclusions - Vacuolated cytoplasm
Immunoprofile	Positive - TTF-1 - Epithelial membrane antigen (EMA) - Napsin A (surface cells) - Cytokeratin 7 Negative - Endothelial markers (CD31, etc.) - Napsin A (stromal cells)

## Data Availability

Not applicable.
